# Metanephric stromal tumor with *BRAF* V600E mutation in an adult patient: Case report and literature review

**DOI:** 10.3389/fonc.2022.993414

**Published:** 2022-10-05

**Authors:** Yuqian Fan, Jingjing Yu, Ming Zhao

**Affiliations:** ^1^Cancer Center, Department of Pathology, Zhejiang Provincial People’s Hospital, Affiliated People’s Hospital of Hangzhou Medical College, Hangzhou, China; ^2^Department of Pathology, Tangshan Gongren Hospital, Tangshan, China; ^3^Department of Pathology, Ningbo Yinzhou Second Hospital, Ningbo, China

**Keywords:** NGS: next-generation sequencing, FISH: fluorescence in-situ hybridization, BRAF V600E mutation, metanephric stromal tumor, metanephric tumors, mesenchymal tumor

## Abstract

Metanephric stromal tumor (MST) is a rare, benign pediatric renal neoplasm of uncertain histogenesis that belongs to the metanephric family of tumors. MST involving adult patients is very uncommon, which could cause significant diagnostic confusions. Recent molecular studies have revealed recurrent *BRAF* mutations in MST in pediatric patients which may serve as powerful diagnostic tools for distinguishing MST from other renal stromal tumors. We present a *BRAF*-mutated MST in an adult patient with a brief review of the pertinent literature. To our knowledge, our case represents to date the sixth report of adult MST and the first adult MST proven to harbor *BRAF* mutation. This is a 41-year-old man who was incidentally identified to have a left renal mass by ultrasonography. He had a 5-year history of hypertension which could be controlled with oral antihypertensive drug. Partial nephrectomy was performed which demonstrated a 2.6-cm, oval, circumscribed mass with a fibrotic and firm texture. Microscopic examination showed a hypocellular, spindle cell neoplasm with entrapped nephrons, within a predominantly fibrous and focally myxoid stroma. Foci of hyalinized stroma surrounding entrapped native renal tubules or blood vessels to form concentric collarettes-like structures, and small-sized arterioles showing angiodysplasia, were observed. Immunostains showed the tumor cells to be diffusely positive for CD34. Fluorescence *in-situ* hybridization analysis was negative for rearrangements involving both the *EWSR1* and *FUS* loci. Targeted next-generation sequencing disclosed a pathogenic mutation of *BRAF* exon15: c.1799T>A (p.V600E). The patient’s hypertension normalized without oral antihypertensive drugs 2 months postoperatively and he was in good status 12 months after the surgery. Our case highlights the diagnostic dilemma of MST occurring in adults and points to the usefulness of molecular detection of *BRAF* mutation for arriving at accurate diagnosis.

## Introduction

Metanephric stromal tumor (MST) is a rare, benign stromal neoplasm unique to the kidney that is thought to be part of a spectrum of metanephric family of tumors which also includes the epithelial lesion metanephric adenoma (MA) and the mixed stromal-epithelial lesion metanephric adenofibroma (MAF) (1). The vast majority of MSTs occur in children in their first three years (2), with only few cases involving adults have been documented (3–6). Recently, several molecular studies have revealed that most pediatric MSTs have a *BRAF* V600E mutation (7, 8). Because of its rarity, the preoperative diagnosis of adult MST is often difficult, and the etiology and clinical biology are largely undetermined. We herein present a MST in a 41-year-old man mimicking a low-grade fibroblastic tumor of the kidney and harboring a *BRAF* V600E mutation by targeted next-generation sequencing. To our knowledge, our case represents to date the sixth report of adult MST and the first adult MST proven to harbor *BRAF* mutation in the literature.

## Case presentation

**Figure 1:**
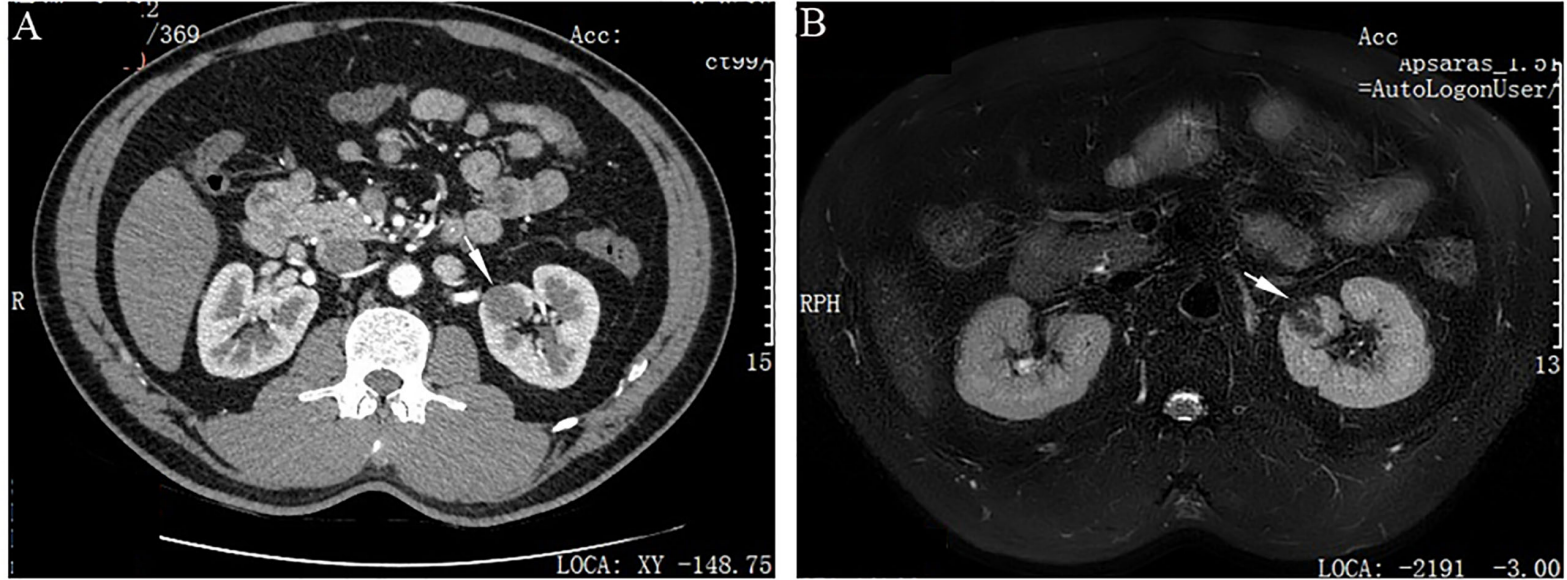
Imaging features of adult metanephric stromal tumor with *BRAF* V600E mutation. **(A)** Computed tomography scan shows a slightly enhanced, well-defined, oval mass (white arrow), in the upper pole of the left kidney. **(B)** Magnetic resonance imaging demonstrates that the mass has low-signal intensity on T2-wighted imaging (white arrow).

A 41-year-old man was incidentally identified to have a left renal mass by ultrasonography for physical examination. His medical history was unremarkable except for a 5-year history of hypertension, which could be controlled with the oral antihypertensive drug hydrochlorothiazide. Workup for 24-hour urine metanephrines and norepinephrine was negative. Computed tomography scan showed a focally low-enhancing, circumscribed, oval mass in the upper pole of the left kidney (**Figure 1A**). A subsequent magnetic resonance imaging revealed that the mass had iso-signal intensity on T1-weighted and low-signal intensity on T2-wighted with enhancement after contrast injection (**Figure 1B**). Neither local invasion nor lymphadenopathy was identified. A benign or low-grade mesenchymal tumor was suspected, and the patient underwent left laparoscopic partial nephrectomy.

**Figure 2:**
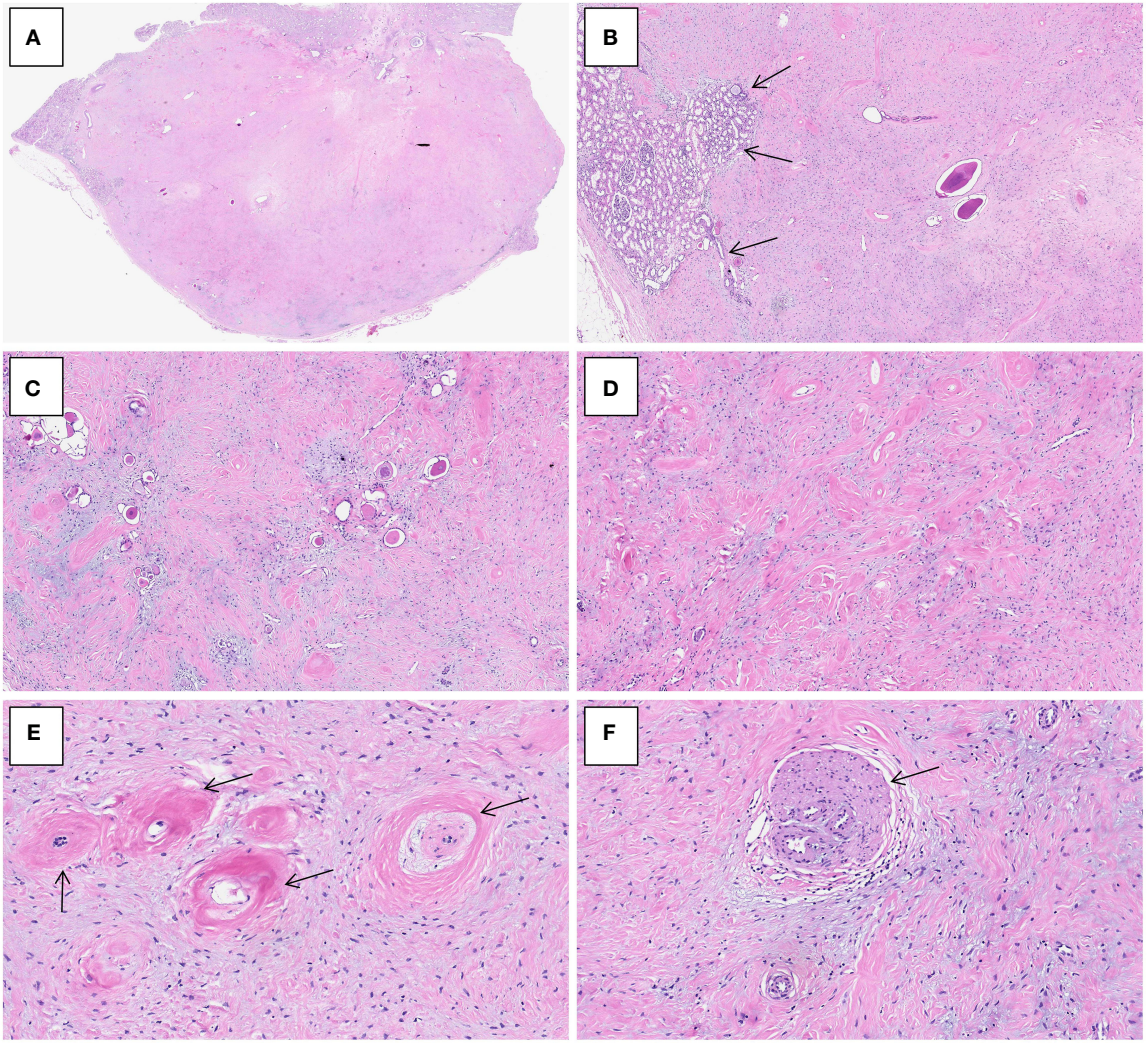
Histological characteristics of adult metanephric stromal tumor with *BRAF* V600E mutation. **(A)** At low power, the tumor is non-encapsulated and has an overall expansile contour. H&E×15. **(B)** Subtly infiltrative border of the tumor with adjacent normal renal parenchyma (arrows). H&E×40. **(C)** Numerous entrapped native renal tubules are noted. H&E×100. **(D)** The tumor is composed of bland, spindle- or stellate-shaped cells, set in a predominantly collagenous and focally myxoid stroma. H&E×100. **(E)** The highly hyalinized stroma encircles and entraps native renal tubules (arrows). H&E×300. **(F)** The tumor induces angiodysplasia within entrapped blood vessels (arrow). H&E×300.

**Figure 3:**
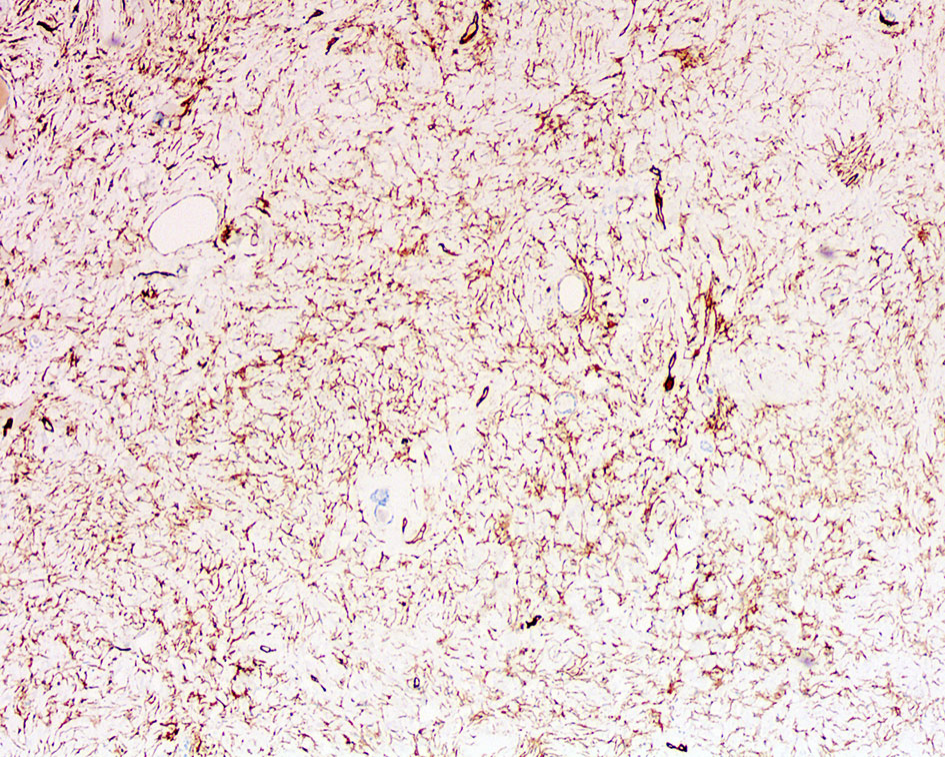
Immunohistochemistry of adult metanephric stromal tumor with *BRAF* V600E mutation. The tumor cells are diffusely and strongly positive for CD34. ×100.

**Figure 4:**
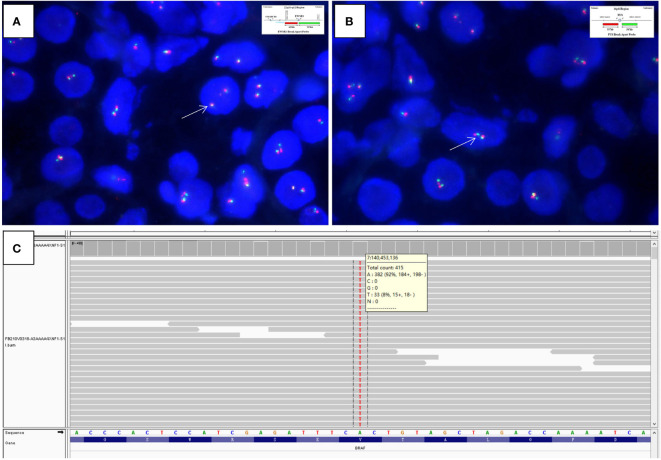
Fluorescence *in-situ* hybridization analysis is negative for rearrangements of both the **(A)** *EWSR1* (×1500, arrows indicate fused green and orange signals) and **(B)** *FUS* (×2000, arrows indicate fused green and orange signals) loci (Insets in A and B indicate schematic diagram of break-apart probes flanking *EWSR1* and *FUS*, respectively). **(C)** Targeted next-generation sequencing discloses the pathogenic mutation of *BRAF* exon15: c.1799T>A (p.V600E) in the tumor, as illustrated by the Integrative Genomics Viewer screenshot.

Grossly, the resection specimen showed a well-circumscribed, nodular mass measuring 2.6×2.0×1.0 cm, centered on the renal cortex of the kidney. On sectioning, the mass was non-encapsulated, firm and fibrotic in consistency and whitish in color. Histologically, the tumor had overall expansile but focally infiltrative, scalloped margins, and numerous entrapped native renal tubules were noted throughout the tumor (**Figures 2A** **–C**). The tumor was composed of hypocellular, bland-appearing, ovoid- to spindle- or stellate-shaped cells, set in a predominantly collagenous and focally myxoid stroma (**Figures 2D** **–F**). Frequently, the highly hyalinized stroma surrounded entrapped native renal tubules or blood vessels forming concentric collarettes-like structures (**Figure 2E**). Occasionally, small-sized arterioles showing angiodysplasia were observed (**Figure 2F**). Mitotic figure was absent. By immunohistochemistry, the neoplastic cells were diffusely positive for CD34 (**Figure 3**), and focally positive for estrogen and progesterone receptors; they were negative for PAX8, S100 protein, STAT6, MUC4, cytokeratin, WT1, and actin. The Ki67 proliferation index was less than 1%. Fluorescence *in-situ* hybridization analysis revealed negative for rearrangements involving both the *EWSR1* and *FUS* loci (**Figures 4A** **, B**). Genetic testing using targeted next-generation sequencing for 425 cancer-relevant genes (GENESEEQ PRIME) disclosed the pathogenic mutation of *BRAF* exon15: c.1799T>A (p.V600E) in the tumor (**Figure 4C**). On the basis of the pathological and molecular genetic features, a diagnosis of adult MST was rendered.

The tumor was completely removed with negative resection margins. The operation was uneventful, and the patient was discharged home on postoperative day 5. As of 2 months postnephrectomy, the patient’s hypertension has normalized without oral antihypertensive drugs and he was in good status 12-month after the surgery.

## Discussion

MST typically occurs in the first years of life (mean: 2 years) with only a few cases documented after 3 years (2). MST affecting adult patients is even rarer with only 6 cases (including the current one) have been described to date (**Table 1**) (3–6). There is no sex predilection with age ranging from 43 to 77 years (mean: 54 years). The clinical presentations are similar to those in pediatric patients, including abdominal pain or mass in 3 cases, hematuria in 1, and hypertension in 2 (3–6). Hypertension as a symptom is uncommonly seen in MST, presenting in less than 10% of pediatric cases (2). The previously reported adult MST with hypertension by McDonald et al. (5) in 2011 was a 55-year-old woman who had a 30-year history of neurofibromatosis type I and new-onset of refractory hypertension. As with our case, the patient’s hypertension normalized in a short period after resection of the tumor. It is believed that the symptom of hypertension may be associated with the tumor’s ability to entrap cortical glomeruli and induce juxtaglomerular cell hyperplasia and subsequent renin secretion (2, 5).

**Table 1:** Summary of adult metanephric stromal tumor reported in the literature.

Case(reference)	Age(years)/Sex	Clinical presentations	Imaging features	Size	Molecular analysis	Follow-up (months)
1 (3)	53/Female	Incidentally identified on workup for other diseases	CT: enhancing mixed solid cystic mass with mural calcifications in the right kidney	5.5-cm	NA	NA
2 (4)	72/Male	Abdominal mass with pain	NA	21-cm	NA	NED (120)
3 (4)	77/Male	Abdominal mass with pain	NA	18-cm	NA	NED (48)
4 (5, 7)*	55/Female	30-year history of neurofibromatosis type I and new-onset of refractory hypertension	CT: complex cystic mass abutting the renal pelvis in the lower pole of the left kidney	2.5-cm	Negative for *BRAF* mutation by RT-PCR	Blood pressure returned to normal status 3-month postnephrectomy
5 (6)	56/Female	Left lower abdominal pain; hematuria	CT: focally low-enhancing, sharply circumscribed, central mass in the upper pole of the right kidney	9-cm	NA	NA
6 Current case	41/Male	Incidentally identified by ultrasonography for physical examination; 5-year history of hypertension	CT: focally low-enhancing, circumscribed, oval mass in the upper pole of the left kidney. MRI: iso-signal intensity on T1-weighted and low-signal intensity on T2-wighted with enhancement after contrast injection	2.6-cm	Positive for mutation of *BRAF* exon15: c.1799T>A (p.V600E) by targeted NGS; negative for rearrangements involving both the *EWSR1* and *FUS* loci by FISH analysis.	Blood pressure returned to normal status 2-month postnephrectomy. NED (8)

*The case in reference 5 was the same to the 7th case in reference 7.

CT, computed tomography; MRI, magnetic resonance imaging; NA, not available; NED, no evidence of disease; NGS, next-generation sequencing; FISH, fluorescence in-situ hybridization.

Adult MSTs exhibited identical histological features to those in pediatric patients. The tumors could be solid or mixed solid and cystic with size ranging from 2.5 to 21 cm (mean: 7.3cm) (3–6). Morphologically, MST is typically a subtly infiltrative tumor composed of variably cellular, bland-looking, spindle cells embedded in a diffusely fibrous to focally myxoid stroma. It characteristically encircles entrapped native renal tubules in a concentric, onion skin pattern and frequently induces angiodysplasia in adjacent blood vessels and/or juxtaglomerular cell hyperplasia in entrapped glomeruli (1, 2). It often shows strong immunoreactivity to antibodies against CD34 and vimentin (2). The molecular basis of MST remains largely unknown until most recently when two separate groups identified mutations in *BRAF* gene (specifically V600E) in 6/7 (86%) and 11/17 (65%) of cases of MST, respectively (7, 8). These findings provide a common consistent genetic alteration that unifies all 3 members of the proposed metanephric neoplasia family, MST, MAF, and MA, given the fact that the latter two entities also frequently harbor *BRAF* V600E mutations (9, 10). For the previously reported cases of adult MST, only 1 had been tested for *BRAF* mutation and was negative (7). To our knowledge, the current case represents to date the first report of adult MST which has been proven to harbor *BRAF* mutation. However, further studies with more cases will be necessary to fully characterize the genetic underpinnings of this rare entity.

Although being rare, it remains a formal possibility that MST may be under-recognized in adults, with some cases have been diagnosed as other adult renal neoplasms that can closely resemble MST with entrapped nephrons. The most useful morphological clues for diagnosing MST include onion-skinning, concentric pattern of tumor cells surrounding entrapped renal tubules, and associated angiodysplasia and juxtaglomerular cell hyperplasia; however, these features maybe only subtle and not well-developed in a subset of cases. The differential diagnostic considerations for MST in adults are quite different from those in pediatric patients because of different clinical settings. For pediatric MST, the most important differential diagnosis is cellular congenital mesoblastic nephroma (CCMN) and clear cell sarcoma of the kidney (CCSK) (2). Both entities typically affect children in less than 3 years and vary rarely occur in adults. However, CCMN usually has a pushing border and is composed of sheets of mitotically-active spindle cells identical to infantile fibrosarcoma. Unlike MST, CCMN lacks expression of CD34 while harbors the characteristic gene fusion involving *ETV6-NTRK3* (11). CCSK is histologically characterized by regular branching capillary vasculature, cords cells with open chromatin, and multiple variants pattern. CCSK shows positivity to BCOR and cyclinD1 but negativity to CD34 by immunohistochemistry (12). Molecularly, most CCSKs have a *BCOR* internal tandem duplication, and small subset have gene fusions involving *YWHAE-NUTM2B* or *BCOR-CCNB3* (13, 14). The main differential diagnosis for adult MST includes sclerosing epithelioid fibrosarcoma (SEF) and solitary fibrous tumor (SFT). Both tumors can rarely occur primarily within the kidney and show bland, spindle to epithelioid cells infiltrating or entrapping native tubules with extensive sclerosis, and positivity for CD34 by immunostain (15, 16). SEF usually shows immunoreactivity for MUC4 and harbors the characteristic gene rearrangements involving *EWSR1*, or less commonly *FUS* (15), which are absent in MST. SFT commonly has prominent HPC-like vessels and demonstrates the characteristic gene fusion of *NAB2-STAT6*, leading to the extensive expression of STAT6 (16); these features are unexpected in MST. Lastly, mixed epithelial and stromal tumor (MEST) of the kidney, which is characterized by biphasic epithelial and stromal components with spindle stroma, glands, and cysts, may also enter into the differential diagnosis of MST. However, MEST mostly occurs in perimenopausal women; the epithelial structures in MEST are more complex and are often lined by hobnail cells, and the stromal cells usually show smooth muscle and mullerian differentiation, which are typically positive for actin, desmin, CD10, and estrogen and progesterone receptors (17). Importantly, as *BRAF* V600E mutation has not been identified in other renal stromal tumors, the presence of this mutation may support the diagnosis of MST in difficult cases (8), just as our case has illustrated.

Adult MST is very rare and accurate diagnosis has important clinical significance. All the reported adult MSTs have had a benign course without recurrence or metastasis (3–6). Excision of the tumor is adequate therapy. The extra-renal angiodysplasia symptom induced by MST, such as hypertension, can relieve after resection of the tumor. Our case highlights the diagnostic dilemma of MST occurring in adults and points to the usefulness of molecular detection of *BRAF* mutation for arriving at accurate diagnosis.

## Data availability statement

The raw data supporting the conclusions of this article will be made available by the authors, without undue reservation.

## Ethics statement

The studies involving human participants were reviewed and approved by the Institutional Review Board Committee of Zhejiang Provincial People’s Hospital, Affiliated People’s Hospital, Hangzhou Medical College. Written informed consent for participation was not required for this study in accordance with the institutional requirements.

## Author contributions

YF, JY, and MZ were involved in conception and design of the work, acquisition, analysis and interpretation of data, drafting the manuscript and revising it critically for important intellectual content and scientific integrity. All authors have read and approved the final manuscript.

## Funding

This work was supported by Zhejiang Provincial Natural Science Foundation (LY21H160052) and Zhejiang Provincial Medicine and Health Research Foundation (2023KY040). The funder did not have any role in the design and conduct of the study, the analysis and interpretation of the data, and preparation of the manuscript.

## Acknowledgments

The authors are grateful to Anbiping Laboratory, Guangzhou, China, for FISH analysis.

## Conflict of interest

The authors declare that the research was conducted in the absence of any commercial or financial relationships that could be construed as a potential conflict of interest.

## Publisher’s note

All claims expressed in this article are solely those of the authors and do not necessarily represent those of their affiliated organizations, or those of the publisher, the editors and the reviewers. Any product that may be evaluated in this article, or claim that may be made by its manufacturer, is not guaranteed or endorsed by the publisher.
